# Steroid lifting method during endoscopic submucosal dissection: A novel strategy for stricture prevention

**DOI:** 10.1002/deo2.403

**Published:** 2024-07-22

**Authors:** Masami Omae, Henrik Maltzman, Miroslav Vujasinovic, Naining Wang, Francisco Baldaque‐Silva

**Affiliations:** ^1^ Department of Upper Abdominal Diseases Karolinska University Hospital Stockholm Sweden; ^2^ Department of Medicine Huddinge Karolinska Institutet Stockholm Sweden; ^3^ Department of Pathology Karolinska University Hospital Stockholm Sweden; ^4^ Department of Gastroenterology Advanced Endoscopy Center Carlos Moreira da Silva Pedro Hispano Hospital Matosinhos Portugal

**Keywords:** Barrett's esophageal neoplasia, complete circumferential resection, endoscopic submucosal dissection, esophageal stricture, steroid lifting method

## Abstract

A 73‐year‐old male patient was referred to us with a long Barrett's esophagus (BE). He had a history of pulmonary embolism under anticoagulant therapy. Esophagogastroduodenoscopy showed a C8M9 BE with no macroscopic lesions. Random biopsies from the BE revealed multifocal high‐grade dysplasia. The case was discussed in a multidisciplinary team conference and the decision for full resection of BE with endoscopic submucosal dissection (ESD) was made. Considering the large ESD resection and the high risk of stricture, we developed a novel preventive technique: the “steroid lifting method” for submucosal injection during ESD. Complete circumferential ESD with en bloc resection was performed using the “steroid lifting method”, without adverse events. Oral liquids were initiated on day 1 and the patient was discharged on day 4. Oral prednisolone (30 mg per day) was started and tapered for a total of 6 weeks. The pathological examination confirmed multifocal high‐grade dysplasia, with radical and curative resection. The patient had neither stricture, dysphagia nor recurrence of Barrett's mucosa at the 2, 6, 12, and 24‐month follow‐up. International guidelines recommend oral prednisolone and triamcinolone injection to prevent stricture formation in large ESD of esophageal squamous cell carcinoma. However, there is no solid data on BE ESD. The risk factors for stricture formation and the optimal preventive management after large BE ESD is not known. The “steroid lifting method” might be an option in this context. Large prospective studies addressing stricture formation and preventive measures on BE ESD are necessary.

## INTRODUCTION

Endoscopic submucosal dissection (ESD) was developed for en‐bloc resection of early gastrointestinal neoplasms. The role of ESD is established in superficial esophageal squamous cell neoplasia, early gastric neoplasia, and some colorectal lesions according to several international guidelines.^1^, ^2^ However, ESD is not yet the standard treatment for Barrett's esophageal neoplasia (BEN). Current guidelines recommend endoscopic resection of visible neoplastic lesions in Barrett's mucosa. Ablation of the remaining Barrett's esophagus (BE) is recommended due to the risk of synchronous or metachronous neoplasia in 11–47% of patients.^1^, ^2^, ^3^ In most Western centers, endoscopic resection of BEN is performed with endoscopic mucosal resection due to its safety profile and feasibility in an outpatient setting. Patients with focal dysplastic lesions in long segment BE need several sessions of both endoscopic resection and ablative treatments. These treatments – endoscopic mucosal resection, ESD, and radiofrequency ablation – may be associated with stricture formation, which affects patients’ quality of life and increases the costs of BE treatment. We developed a novel strategy for stricture prevention, the “steroid lifting method” that aims to reduce stricture formation in large BE ESDs. We present a case of complete circumferential resection of multifocal high‐grade dysplasia (HGD) using this novel technique to prevent stricture formation during the 24‐month follow‐up period.

## CASE REPORT

**FIGURE 1:**
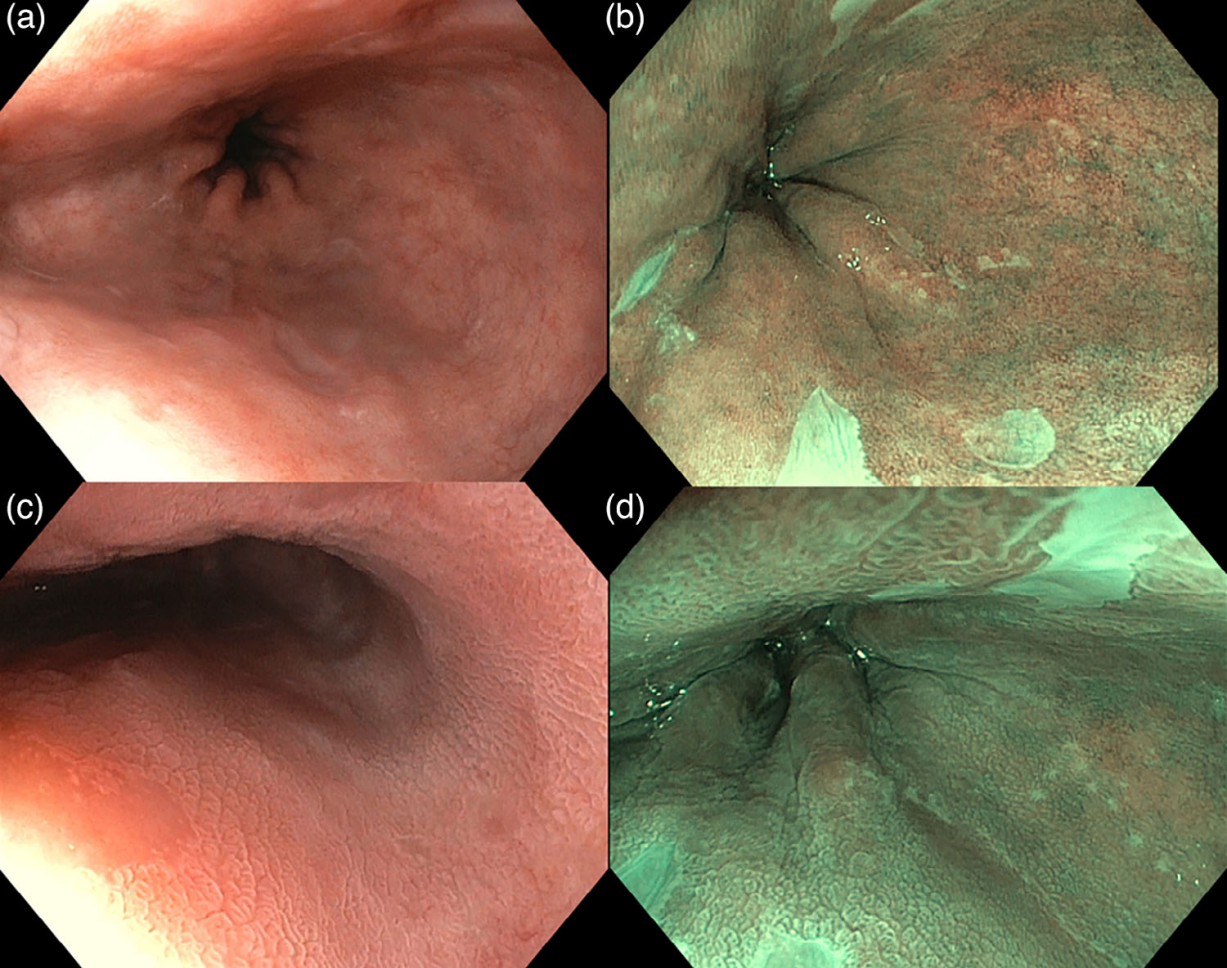
Endoscopic evaluation before endoscopic submucosal dissection. (a) White‐light imaging showing Barrett's esophagus (C8M9) without any visible lesions. (b) Narrow band imaging shows no visible lesions in Barrett's esophagus. (c) White‐light imaging with the application of acetic acid showing no visible lesions. d. Narrow band imaging with applications of acetic acid showing no visible lesions.

A 73‐year‐old male patient with a history of pulmonary embolism under anticoagulant therapy was referred to us due to an long segment BE (C8M9) with positive biopsies for HGD. A new esophagogastroduodenoscopy (EGD) was performed at our center using white light, narrow band imaging, and chromoscopy with acetic acid. No macroscopic lesions were detected (Figure 1). However, random biopsies confirmed the presence of multifocal HGD along the BE.

This case was discussed at a multidisciplinary team conference and the decision for full resection of BE with ESD was made. The procedure was planned after the patient's signed informed consent.

### The steroid lifting method

**FIGURE 2:**
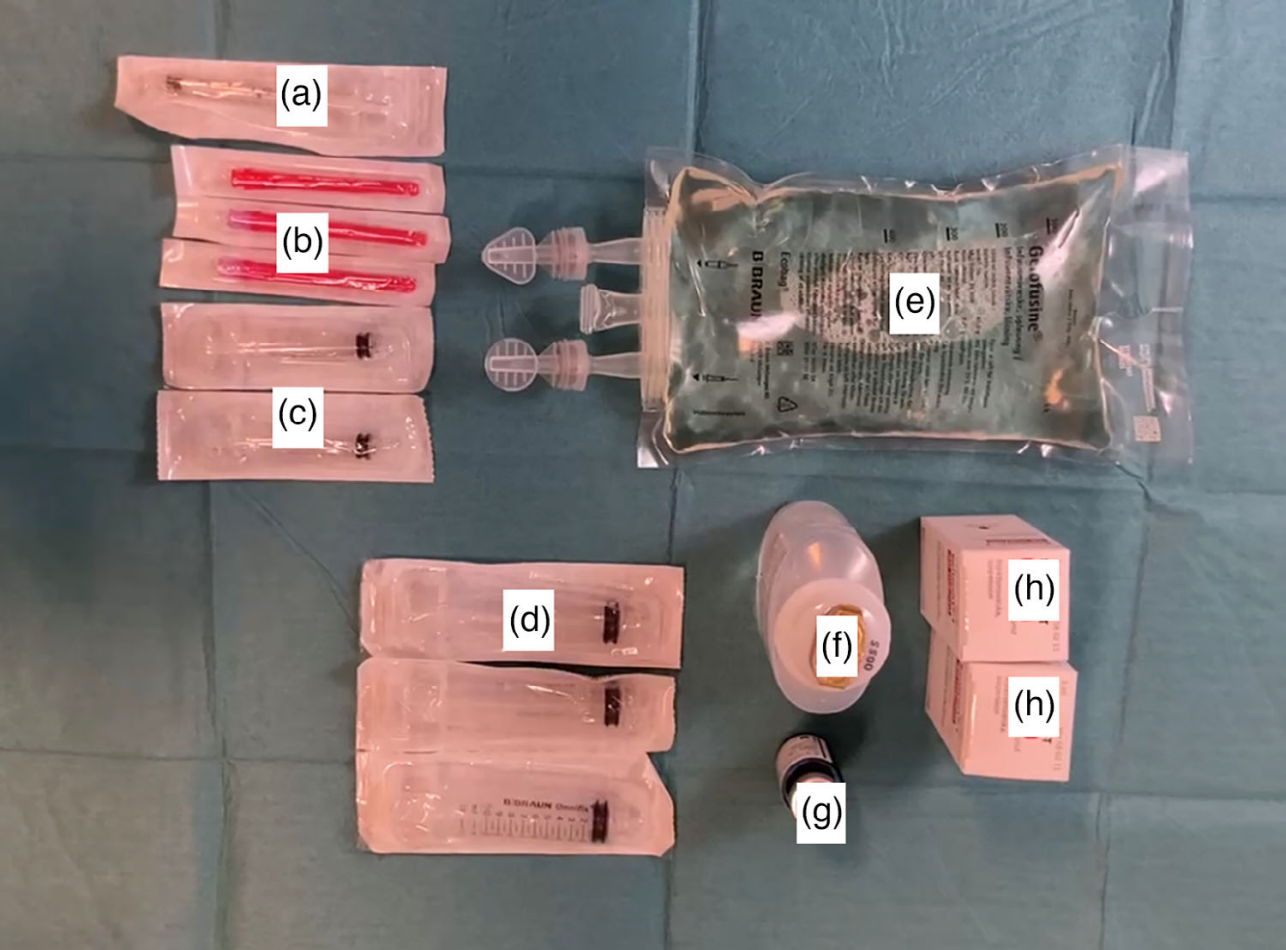
Material needed for the steroid lifting methods. (a) 1 mL syringe. (b) 18‐gauge needle. (c) 5 mL syringe. (d) 10 mL syringe. (e) Gelofusine. (f) Saline solution. (g) Indigo carmine. (h) Triamcinolone acetonide. One milliliter of triamcinolone acetonide 40 mg/mL (h) is aspirated with an 18‐gauge needle (b) using a 1 mL syringe (a) and then mixed with 3 mL of saline solution (f) in a 5 mL syringe (c). (referred to as syringe A). Nine milliliters of Gelofusine (e) are then mixed with 1 mL of the mixture from syringe A in a 10 mL syringe (referred to as syringe B). (d) This mixture is used as the lifting solution during the endoscopic submucosal dissection procedure.

Succinylated gelatin (Gelofusine; B. Braun Medical AB) is a colloidal plasma volume substitute commonly used in fluid resuscitation. Compared to hyaluronic acid, it is cheaper, more available in the West, and easier to mix with solutions like indigo carmine, adrenaline, and steroids. It can also be used through ESD needles in the “water jet.” In this method, 500 mL of succinylated gelatin is mixed with 3 mL of indigo carmine and used for submucosal injection. One milliliter of triamcinolone acetonide (Kenakort; Bristol‐Myers Squibb, 40 mg/mL) is mixed with 3 mL saline solutions in a 5 mL syringe (referred to as syringe A). Nine milliliters of Gelofusine are then mixed with 1 mL of the mixture from syringe A, in a 10 mL syringe (syringe B). This mixture is used as the lifting solution during the ESD procedure (Figure 2).

### Endoscopic submucosal dissection

**FIGURE 3:**
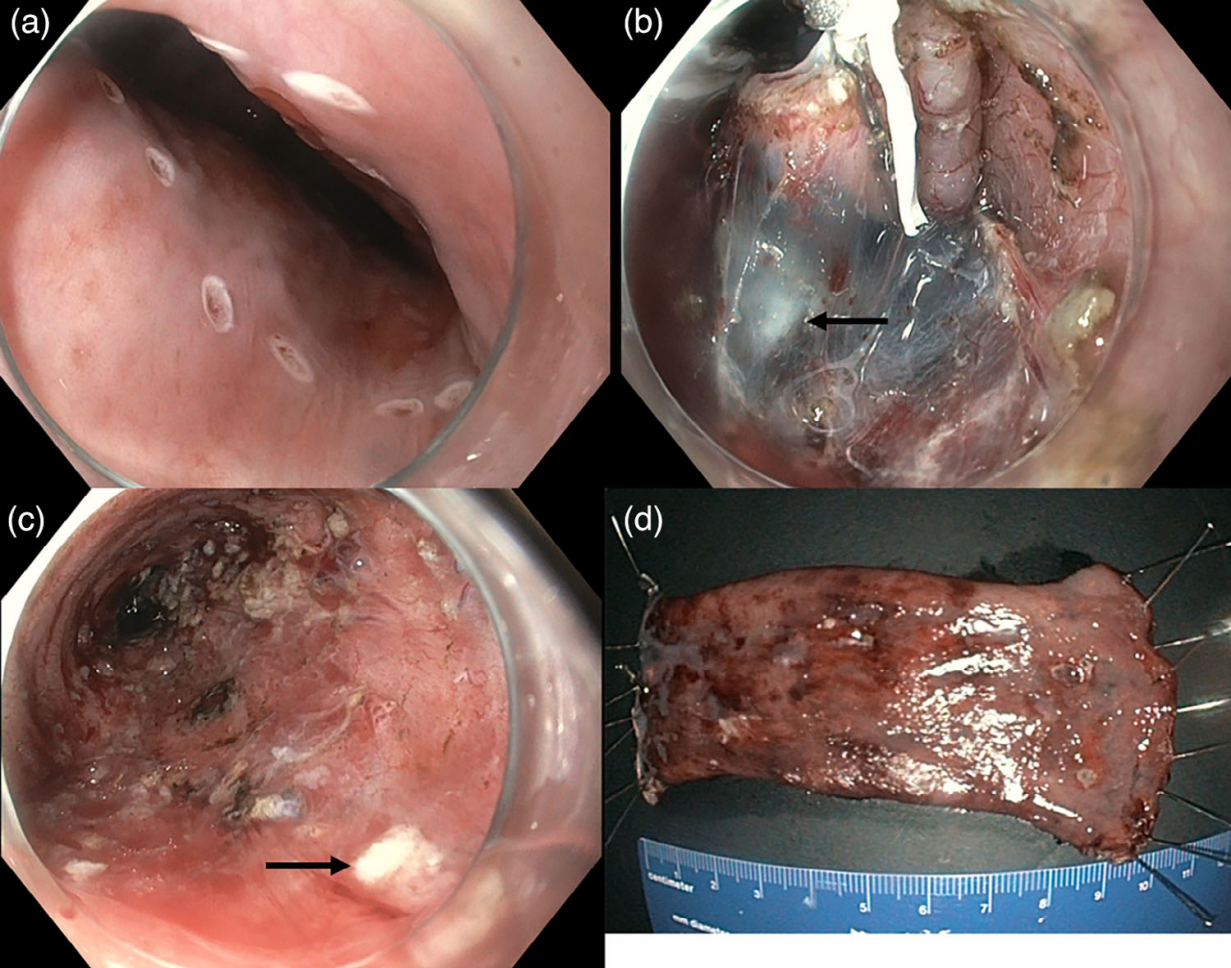
The endoscopic submucosal dissection procedure. (a) The proximal side of the lesion with markings. (b) A clip‐and‐thread technique was used. Black arrow shows a whitish area corresponding to triamcinolone in the submucosal layer. (c) The mucosal defect after endoscopic mucosal dissection. Black arrow shows a whitish area corresponding to triamcinolone in the submucosal layer. (d) The resected specimen was 108 × 45 mm.

**FIGURE 4:**
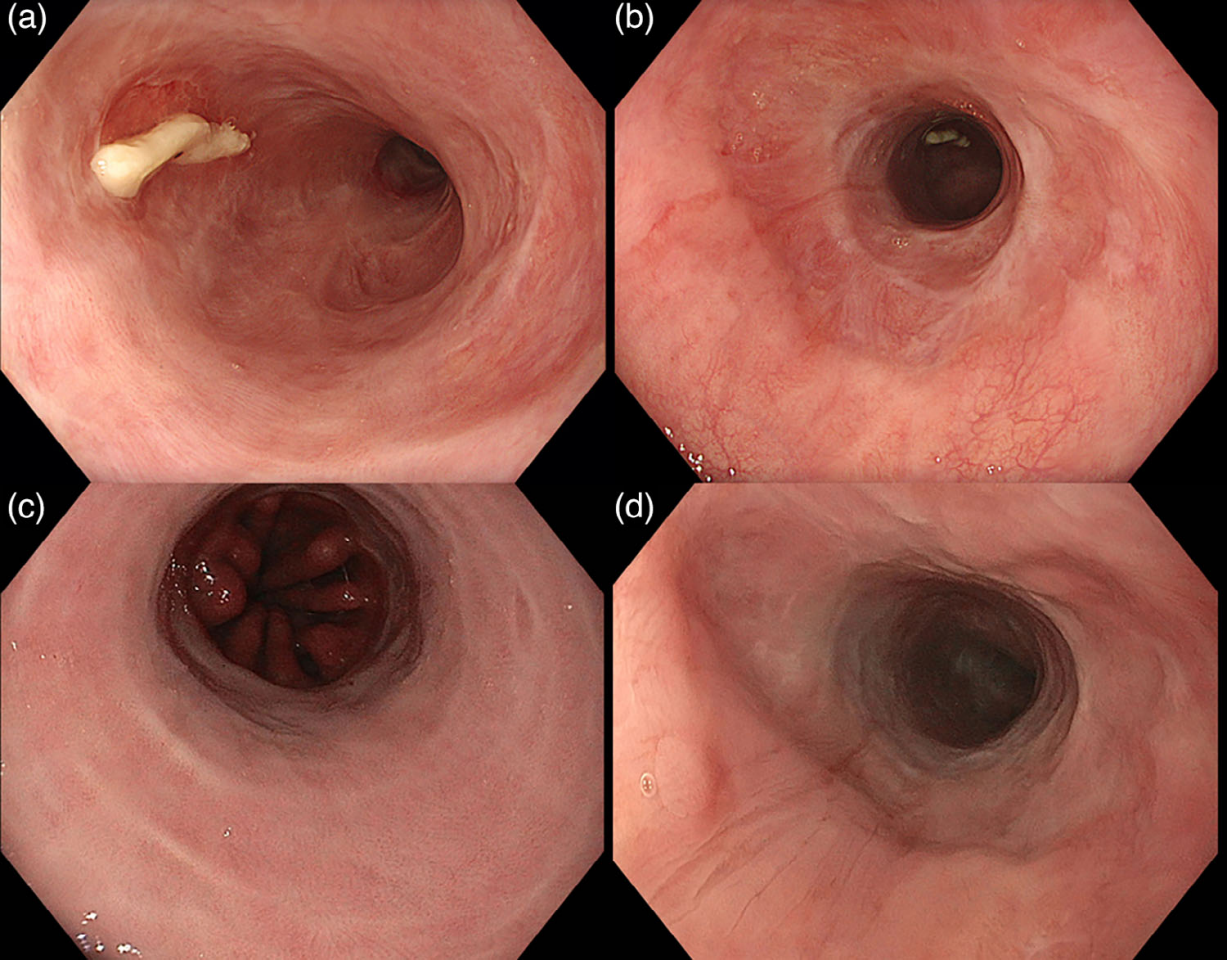
Follow‐up endoscopy at 2 and 6 months follow up. (a, b) WLI showing fibrin‐coated ulcer without any signs of stricture and recurrence of Barrett's esophagus at 2 months. (c, d) White‐light imaging showing an endoscopic submucosal dissection scar without any signs of stricture or recurrence of the Barrett's esophagus at 6 months follow‐up.

Before ESD, marking dots were placed in the proximal and distal BE margins using the dual knife (KD‐650 L; Olympus). The steroid lifting solution was then used for submucosal lifting and mucosal incision was performed on the distal and proximal side margins. The same solution was used during the ESD when needed for additional submucosal lifting. Two tunnels were created during ESD using the clip‐and‐thread technique.^4^ Complete en‐bloc resection was obtained without complications (Figure 3). A total of 200 mg triamcinolone acetonide was used in the lifting solution during the ESD. The retrieved specimen was 108 × 45 mm in size. The liquid diet was resumed on day 1 and the patient was discharged from the hospital on day 4. Oral prednisolone was given for 6 weeks, starting at the dose of 30 mg per day. The pathological examination confirmed multifocal HGD with radical (R0) and curative resection. Follow‐up EGD showed no sign of stricture and no recurrence of the BE at 2, 6, 12, and 24 months. (Figure 4).

## DISCUSSION

Endoscopic submucosal dissection enables en‐bloc resection of neoplastic lesions and has been widely used along the gastrointestinal tract except for BEN. Initial reports of ESD for BEN were discouraging due to the high rate of positive lateral margins and a significant rate of complications. More recent studies have shown good efficacy of ESD for BEN, with lateral R0 rates of 75%–90%.^5^ In our early series of neoplastic BE treated with ESD, the rates of en bloc, R0 and curative resection were 99%, 86%, and 72%, respectively.^6^ However, ESD of BEN may be associated with adverse events such as stricture formation in the esophagus during the healing process. This complication is now the primary factor preventing the widespread use of ESD for BEN. The risk of stricture is high in previous studies, with larger specimens and a greater percentage of resected lumen circumference further increasing this risk.^7^

With the available options, stricture formation after esophageal ESD represents a big burden for the patients, medical staff, and health services. The most commonly used strategies to prevent esophageal strictures are corticosteroid therapy, particularly oral prednisolone, and local triamcinolone injection. However, there are some risks associated with systemic steroid use. These risks include hyperglycemia, cytomegalovirus enterocolitis and other infections, cardiac arrhythmia, hypokalemia, and psychological disorders. Triamcinolone injection also carries a risk of perforation, necrosis, and bleeding, with severe outcomes in some cases. Additionally, triamcinolone injection is traditionally performed after ESD and is time‐consuming. Furthermore, neither oral prednisolone nor local triamcinolone injection has been shown to be effective in circumferential ESD.^8^

Other preventive strategies have been described in small series, such as endoscopic metal stents, injection of botulinum toxin type A, oral administration of tranilast (N‐[3,4‐dimethoxycinnamoyl]‐anthranilic acid), tissue‐shielding methods with polyglycolic acid and oral budesonide.^9^ It is not known how these preventive strategies impact results as BEN ESD is not widely performed in the West, resulting in a lack of data.

There are some advantages of our novel prevention technique. The steroid lifting method is performed directly during ESD and uses conventional devices. It has a topical effect, and no additional procedure time is required afterward. The treatment is also fast to deliver, has low costs, and may be associated with a lower risk of perforation compared to the current triamcinolone injection method because the submucosal layer can be identified during ESD. We argue that this method may be more effective for superficial ESD layers, as triamcinolone can be retained in the ulcer, but less effective for deeper layers often involved in malignant lesions. However, this method ensures that the steroid spreads across different layers of the submucosa, allowing it to reach free submucosal tissue regardless of tumor depth. Injecting steroids before the incision means they will be present not only in the ulcer after ESD but also in the surrounding healthy tissue. A whitish area can also be detected during ESD (Figure 3b,c, black arrows) as a result of the steroid injection.^10^ However, due to a lack of data, it is unclear if this method is superior to the current steroid injection technique. Additionally, in this case, the impact of the steroid lifting method on stricture prevention remains uncertain as the patient also took systemic steroids. There is a need to further evaluate the risk factors for stricture formation and identify the best strategies for its prevention.

Herein, we present a new technique for the prevention of strictures that is easy to deliver, cheap, and safe. The procedure was performed without complications in our patient, who had a 9 cm long segment BE and the width of the resected specimens of BE was 45 mm. However, prospective studies are needed to test and validate this novel strategy.

## CONFLICT OF INTEREST STATEMENT

None.

## ETHICS STATEMENT

Not applicable.

## PATIENT INFORMED CONSENT

Complete written informed consent was obtained from the patient for the publication of this study and accompanying images.
